# ILs and MMPs Levels in Inflamed Human Dental Pulp: A Systematic Review

**DOI:** 10.3390/molecules26144129

**Published:** 2021-07-07

**Authors:** Konstantina Kritikou, Maria Greabu, Marina Imre, Daniela Miricescu, Alexandra Ripszky Totan, Marian Burcea, Iulia-Ioana Stanescu-Spinu, Tudor Spinu

**Affiliations:** 1 Department of Biochemistry, Faculty of Dental Medicine, Carol Davila University of Medicine and Pharmacy, 8 Eroilor Sanitari Blvd, Sector 5, 050474 Bucharest, Romania; constantinak8@gmail.com (K.K.); maria.greabu@umfcd.ro (M.G.); daniela.miricescu@umfcd.ro (D.M.); iulia.stanescu@umfcd.ro (I.-I.S.-S.); 2 Department of Complete Denture, Faculty of Dental Medicine, Carol Davila University of Medicine and Pharmacy, 8 Eroilor Sanitari Blvd, Sector 5, 050474 Bucharest, Romania; marina.imre@umfcd.ro; 3 Department of Ophthalmology, Faculty of General Medicine, Carol Davila University of Medicine and Pharmacy, 8 Eroilor Sanitari Blvd, Sector 5, 050474 Bucharest, Romania; 4 Department of Fixed Prosthodontics and Occlusion, Faculty of Dental Medicine, Carol Davila University of Medicine and Pharmacy, 17-23 Calea Plevnei, 010221 Bucharest, Romania; tudor.spinu@umfcd.ro

**Keywords:** dental pulp, inflammation, interleukins, matrix metalloproteinases, enzyme linked immunosorbent assay, western blot

## Abstract

A wide range of mediators are released from the pulp tissue because of bacterial invasion which causes inflammation. Interleukins (ILs) and matrix metalloproteinases (MMPs) have a leading role in initiating and spreading of inflammation because of their synergic action. Biomarkers such as ILs and MMPs can be identified via several methods, establishing the inflammatory response of the dental pulp. The aim of this systematic review is to evaluate the levels of ILs and/or MMPs in human dental pulp. PubMed, OVID, Cochrane, Scopus, Web of Science and Wiley online library databases were searched for original clinical studies. After applying inclusion and exclusion criteria, a quality assessment of studies was performed based on a modified Newcastle-Ottawa scale. In the review were included articles that evaluated the presence of ILs and/or MMPs in pulp tissue using enzyme-linked immunosorbent assay (ELISA) or western blot or multiplex assay. Six articles were included in the present synthesis. Although various diagnostic methods were used, statistically significant higher levels of ILs and/or MMPs were mostly found in the experimental groups compared to healthy pulp samples. The biomarkers studied can be a promising tool to evaluate pulp tissue health or even in pulpitis treatment.

## 1. Introduction

**Figure 1:**
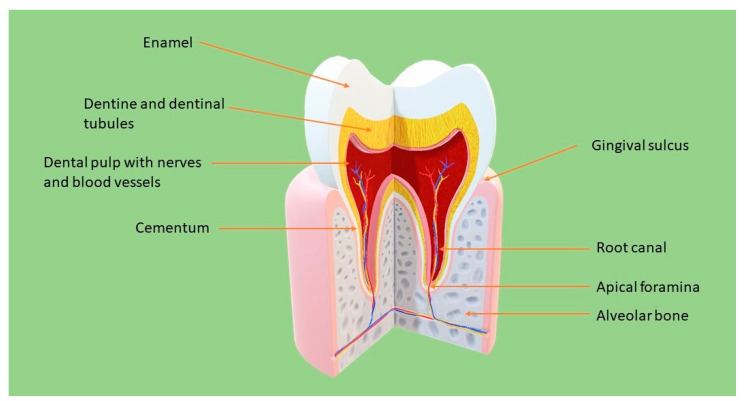
Molar anatomy.

Pulp tissue has its origin in the ectomesenchyme and derives from neural crest cells. Neural crest cells proliferate and are condensed, leading to the formation of mesenchymal dental papilla. Dental pulp (Figure 1) is located in a rigid chamber which offers mechanical support and protection from the oral environment rich in microorganisms. Pulp structure is based on collagen molecules, proteoglycans, fibronectin and different types of glycoproteins [1]. In the peripheral zone of the pulp exists a layer of highly specialized cells, the odontoblasts [2]. Alongside these cells exist undifferentiated mesenchymal cells which will become odontoblast-like cells forming dentine if stimulated [1]. Other cellular components of dental pulp are immune cells that participate in the initial recognition and the following processing of antigens triggering pulp defence. Dental pulp’s immune cells are T cells, T helper and T cytotoxic, dendritic cells and macrophage-like cells. Innervation of pulp tissue is provided mainly by myelinated A-fibers and unmyelinated C-fibers. Nerve fibers are disposed along radicular to coronal pulp where they form a plexus in the subodontoblast region. Changes in the blood flow modulate sensory nerve function. For example a decrease of blood flow supresses A-fiber activity [3]. Dental pulp’s rich microvasculature and sensory nerve innervation make it a unique tissue.

Caries and fractures are the two main reasons that can affect the integrity of the pulp chamber, creating pathways for microorganisms and their metabolites to enter the pulp [1]. The response of the pulp to these afflictions is inflammation, which can spread to the surrounding tissues and alveolar bone [4]. Each type of short- or long-term burden will have a different result on the pulp, such as acute or chronic inflammation or necrosis of the pulp tissue [1]. Acute inflammation is often caused by short-term irritants like cavity preparation which can be followed by repair of the tissue as long as the irritant’s action does not persist. On the other hand, long-term irritants like caries cause chronic inflammation of the pulp which frequently leads to necrosis.

Pulp inflammation or pulpitis is a clinical and histologic term that indicates inflammation of the pulp tissue. It can be described clinically as reversible or irreversible and histologically as acute and chronic. Acute and chronic terms can also caracterize the inflammation according to clinical manifestations. An acute inflammation is associated with an intense, sharp and progressive pain with a duration from a few hours to 24–48 h and that leads the patient to seek emergency help. The pain is diffuse and often the stimulus is represented by heat or cold. Frequently, the patient cannot localize exactly the tooth. On the other side, a chronic pulp inflammation can be manifested by numerous episodes of dull pain which responds to analgesics, and as a result, the patient does not seek emergency medical care; the pain is often located and appears when chewing [5].

Depending on the condition of the pulp chamber and dental pulp, chronic inflammation can be closed (internal granuloma) or open (ulcerative form or hyperplastic form = pulp polyp) [6,7]. Irreversible inflammation can be symptomatic or asymptomatic and the diagnosis is based mainly on patients’ signs and symptoms. The term irreversible denotes that pulp cannot heal and must be removed from the endodontic space. Irreversible pulpitis can be associated with lingering and spontaneous pain (symptomatic) or with the absence of clinical symptoms (asymptomatic) [4]. On the other hand, when inflammation is reversible the suitable treatment should promote healing, triggering a decrease of the inflammatory reaction [8].

Pulp inflammatory reaction is initiated by complement activation [4]. Bacterial invasion in dental pulp leads to an increase of monocytes and polymorphonuclear leukocytes (PMNs) population. As inflammation progresses, primed macrophages activate CD4^+^ lymphocytes, triggering cell-mediated immune response [8].

In response to bacterial invasion, cells like macrophages, B and T cells and monocytes release cytokines such as interleukins (ILs) in order to mediate the inflammatory process [9]. Innate immune cells can secrete ILs such as IL-1, IL-6, IL-10, IL-12 and IL-18 with multiple roles; e.g., IL-1 induces macrophage and lymphocyte stimulation, while IL-6 activates B cells [10] and IL-10 inhibits macrophages and dendritic cells’ functions [11].

**Figure 2:**
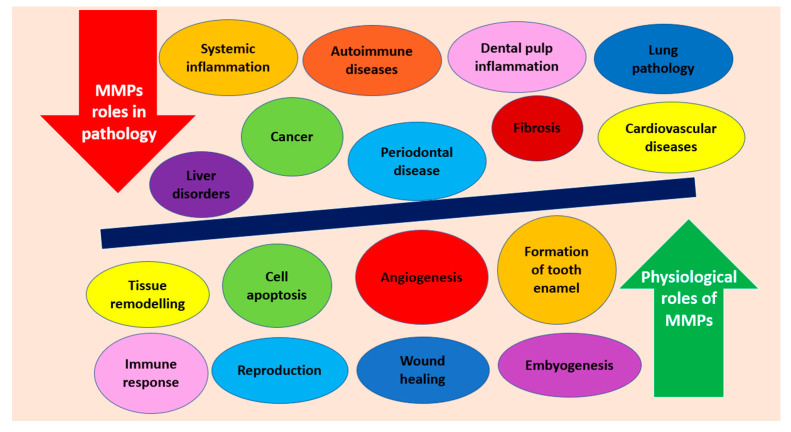
MMPs’ representative roles in physiological and pathological conditions.

Matrix metalloproteinases (MMPs) are a group of Zn-dependent endopeptidases [12]. They can degrade extracellular matrix (ECM) protein components such as basement membrane collagen, proteoglycans, fibronectin and laminin. Tissue inhibitors of metalloproteinases (TIMPs) and nonspecific protein inhibitors such as α_2_-macroglobulin maintain the balance regarding the degenerative potential of MMPs [13]. MMPs can be synthesized by odontoblasts but also by PMNs, macrophages and plasma cells. Uncontrolled activity of MMPs can contribute to excessive tissue destruction and spread of inflammation [14]. The most important molecular roles of MMPs are mentioned in Figure 2.

ILs and MMPs can act synergically in the pathogenesis of dental pulp inflammation. Elevated levels of IL can stimulate pulp cells to produce and secrete MMPs. MMPs can degrade the ECM present in dental pulp but also can destroy newly formatted predentin, which can lead to inhibition of dentinogenesis [15]. Various ILs and MMPs were found in inflamed dental pulp compared to healthy samples with inconsistent results.

Cytokines such as IL-1 can induce mineralized tissue resorption through the stimulation of MMPs synthesis, activation and secretion by the host cells present in the inflamed tissue [15]. Furthermore, IL-1 downregulates protein formation, such as alkaline phosphatase and collagen, which are necessary for extracellular matrix formation.

A better understanding of the underlying roles of ILs and MMPs interrelations in dental pulp inflammation could provide new tools of diagnosis of this group of dental pathology, leading to new perspectives in pulpitis treatment, especially when apexogenesis is desired. Consequently, the aim of the present systematic review is to evaluate the amounts of ILs and/or MMPs in clinically healthy and inflamed dental pulp tissues.

## 2. Materials and Methods

A literature review was performed according to PRISMA guidelines [16].

### 2.1. Selection Criteria

The following criteria were taken into consideration: population was represented by human inflamed pulp samples (acute, chronic, reversible, irreversible inflammation) in response to caries’ progression. The control group was represented by clinically healthy (normal) human pulp samples. The quantitative levels of ILs and/or MMPs were tested using enzyme-linked immunosorbent assay (ELISA), western blot and multiplex assay.

### 2.2. Literature Search

The research was conducted during May 2021 without language restrictions. The electronic databases OVID (last search on 4 May 2021), PubMed (last search 5 May 2021), Web of Science Core Collection (5 May 2021), Wiley Online Library (5 May 2021) and SCOPUS (8 May 2021) were searched using the following MeSH terms: [interleukins AND “dental pulp inflammation”] OR [interleukins AND pulpitis] OR [“matrix metalloproteinases” AND “dental pulp inflammation”] OR [“matrix metalloproteinases” AND pulpitis]. All search strategies are mentioned in Supplementary Material (Table S1). One limitation of the present systematic review may be that the research was performed using only databases in English.

### 2.3. Study Selection

**Figure 3:**
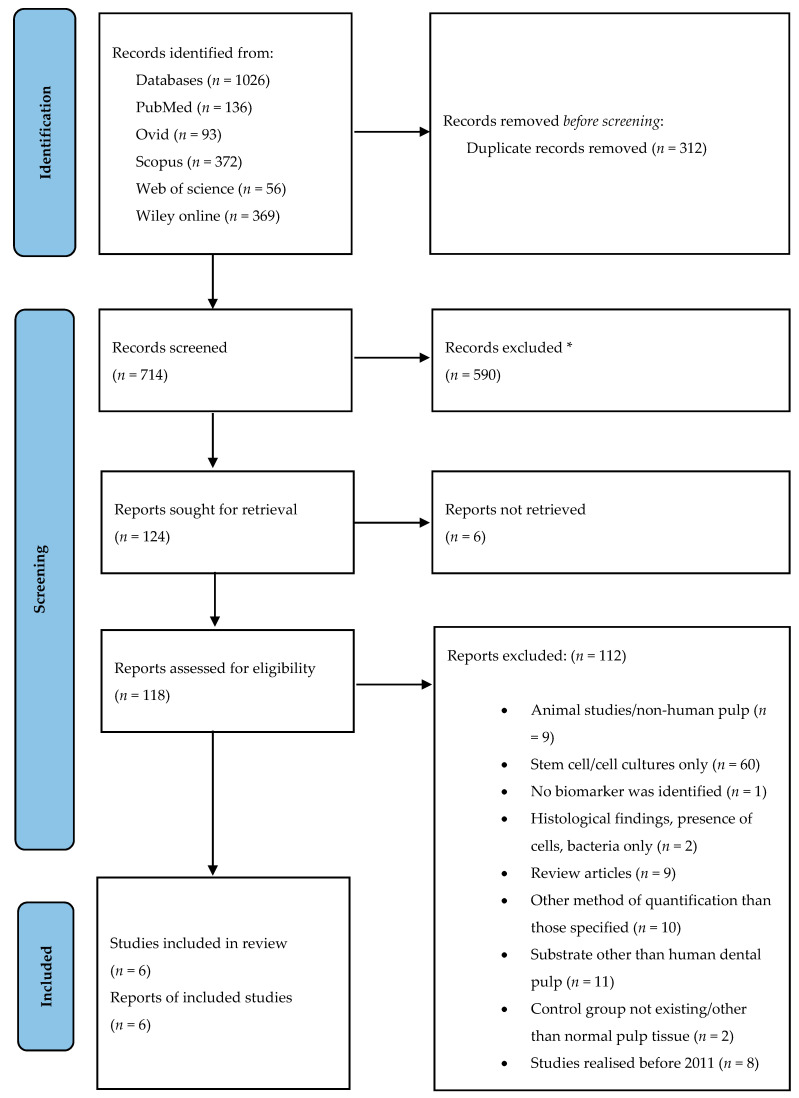
PRISMA outline. Identification of studies via databases. * All records were excluded manually; we did not use any automation tools. From: Page M.J., McKenzie J.E., Bossuyt P.M., Boutron I., Hoffmann T.C., Mulrow C.D., et al. The PRISMA 2020 statement: an updated guideline for reporting systematic reviews. BMJ 2021;372: n71, doi:10.1136/bmj.n71. For more information, visit: http://www.prisma-statement.org/ (accessed on 10 May 2021).

A PRISMA outline (Figure 3) was used to conduct this systematic review [16]. A total of 1026 articles were initially identified using 5 electronic databases (Table S2). We did not find new studies via other methods. A total of 312 duplicate records were removed before screening. Endnote’s duplicate identification strategy was used to remove all duplicates and then this was performed mannually by reviewer T.S.; we did not use any automation tools to exclude articles.

A total of 714 records remained for screening after the removal of duplicates. The titles and abstracts of the references were screened and 590 records, involving a different subject, were excluded. A total of 124 reports were sought for retrieval with 118 of them being considered appropriate and reviewed independently as full texts by 2 reviewers (T.S. and I.-I.S.-S.). We could not retrieve 6 reports (Table S3). In the case of disagreement consensus was reached discussing with a third reviewer (M.G.). After full text screening we decided to limit the included studies to those realised and published in the last 10 years since most of the potential eligible studies were already included in other systematic reviews with a similar topic [17,18,19]. Then, 112 reports were excluded for one of the following reasons: (a) animal studies/non-human pulp studies; (b) stem cell/culture studies only; (c) no biomarker was identified; (d) histological results, presence of cells, bacteria only; (e) review articles; (f) other diagnostic methods than those specified in the inclusion criteria; (g) substrate other than human dental pulp; (h) control group not existing/other than normal pulp tissue; (i) studies published before 2011. Details about excluded studies are mentioned in Supplementary Material (Table S4). Publications which met inclusion criteria were included in the study (*n* = 6).

### 2.4. Data Collection and Quality Assessment

All the data included in this review were extracted by T.S. and I.-I.S.-S. from the selected studies. The following variables were collected in an electronic protocol using Microsoft Excel: authors and year of publication, aim of the study, clinical diagnosis (healthy, reversible inflammation, irreversible inflammation, chronic open and closed inflammation of the pulp) and number (*n*) of specimens in control and experimental group, number (*n*) and age of subjects included in the study, type of identified biomarker, diagnostic method, comparison between experimental and control group and existence of statistically significant differences between them. A narrative synthesis of the data was performed.

Quality assessment of the included studies was performed by M.G. using the Newcastle-Ottawa Quality Assessment scale adapted for cross-sectional studies [20,21]. Each study was judged on the selection of the study groups, the comparability of the groups and the ascertainment of the outcome based on a “star system” (Table S5). A score of 0 matched with a study with the lowest quality while a score of 10 rates a study with the highest quality. Very good studies are those rated with 9–10 stars, good studies with 7–8 stars, satisfactory studies with 5–6 stars while unsatisfactory studies are those assessed with 0–4 stars.

## 3. Results

### 3.1. Studies Included

For our review we have chosen six studies (Table 1). Our focus was to include studies based on protein levels of ILs and/or MMPs in normal and inflamed human dental pulp using ELISA, western blot and multiplex assay as selected tests.

### 3.2. Main Analyte

The systematic review was based on studies that analysed human dental pulp tissue for measurement of ILs and/or MMPs levels. Healthy pulp tissue was obtained after extraction of permanent teeth (impacted mature and immature rooted [22], impacted caries-free [23], third molars [25] and premolars [26]) for orthodontic purposes. After extraction, the teeth were sectioned, split with an elevator, and the pulp tissue was removed and stored until analysis (refrigerated at −80 °C).

Healthy pulp extraction for prosthetic purposes from permanent teeth was performed in two of six studies [6,24] with only one study [24] mentioning the method: samples were removed with a sterile excavator and barbed broach. One of the studies also used as control group healthy pulp tissue from extracted primary teeth with prolonged retention [26]; pulp was collected after cutting and splitting of teeth.

Regarding the study group, two of the included studies were based on pulpectomy for the collection of reversible and irreversible inflamed pulp tissue from permanent teeth [22,24]. Pulpectomy was also the method of election for chronic closed and open inflamed pulp tissue [6]. One of the studies [23] analysed reversible inflamed pulp tissue extracted from permanent teeth for prosthetic purposes. The same study [23] also used irreversible inflamed pulp tissue without mentioning the method of extraction. Endodontic treatment and pulp extraction from chronically inflamed permanent teeth without further details about the method of collection was mentioned in one study [25]. A single study included experimental groups with irreversibly inflamed pulp tissue from permanent teeth and reversibly and irreversibly inflamed pulp tissue (as an experimental group without differentiation) from primary teeth; pulp removal was realised after extraction, sectioning and fracture of the teeth [26].

### 3.3. Diagnostic Tests

We chose to limit the present systematic review to studies which investigated the levels of the biomarkers, using ELISA, western blot and multiplex assay as selected tests. ELISA was used as a diagnostic method in four of the six [6,22,24,25] included studies, while multiplex assay [23] and western blot [26] were each used to detect the level of the chosen biomarkers in one study.

### 3.4. Biomarkers Identified and Types of Inflammation

ILs and/or MMPs levels were quantitatively analysed in the included studies. A total of 14 biomarkers were identified in six studies. ILs were the biomarkers of election in three studies [6,22,23]. MMPs were analysed in two studies [25,26], while both the levels of ILs and MMPs were determined in one study [24].

Experimental groups in the selected studies were represented either by reversible, irreversible or chronic dental pulp inflammation (chronic open inflammation, chronic closed inflammation or without mentioning the type). Of all the studies, only two confirmed their clinical diagnosis with histologic examination [22,23].

IL-1β and IL-6 were each investigated in two studies; IL-1β was determined in experimental groups consisting of reversibly and irreversibly inflamed pulp tissue [23] and chronically closed and open inflamed pulps [6] versus healthy pulp tissue. IL-6 was detected in inflamed pulp samples with reversible and irreversible inflammation, both in studies with and without differential diagnosis between the types of pulpitis [22,23]. IL-8 was investigated in three different studies: in inflamed pulp without differentiation between reversible and irreversible inflammation [22], in inflamed pulp with differentiation between reversible and irreversible inflammation compared to normal pulp [23] and irreversibly inflamed pulp tissue versus normal pulp tissue [24]. Inflamed pulp (with a differential diagnosis of reversible or irreversible inflammation) was analysed for the presence of IL-1α, IL-rα, IL4, IL-7, IL-12p40, IL-13 and IL-15 [23].

The levels of MMP-8 were measured in irreversibly inflamed pulp tissue [24] and in chronically inflamed pulps versus healthy pulp tissue [25]. MMP-9 was the only biomarker investigated in healthy and inflamed pulps from temporary and permanent teeth (healthy versus reversibly and irreversibly inflamed pulps, healthy versus irreversibly inflamed pulps) [26]. MMP-1 and MMP-13 levels were measured in chronically inflamed pulp tissues and compared to normal, healthy pulp tissue [25].

### 3.5. Comparison and Statistical Significance

Almost all studies presented statistical significance for the results among the tested groups. Inflamed pulp tissue compared to healthy pulps reported significant higher levels of mediators in all studies with some exceptions: levels of IL-15 in the irreversibly inflamed pulp tissue group were higher compared to normal pulps, but non-significant, and levels of IL-7, IL-8 and IL-13 were elevated in healthy pulps compared to experimental group with statistical significance [23]. Statistically significant results were also observed in the chronic pulpitis group compared to healthy, as well with some exceptions: healthy pulp tissue showed higher levels for the selected markers compared to the experimental group represented by open chronic inflammation, without statistical significance and chronic (open and closed) inflamed pulps manifested elevated levels compared to healthy pulps, however without statistically significant results [6].

When comparing reversibly inflamed to irreversibly inflamed pulps the obtained results weren’t statistically significant [23]. On the other hand, chronic closed pulpitis group presented significant elevated levels of markers compared to chronic open pulpitis group [6].

### 3.6. Quality of the Studies

The scores of the included studies ranged between 4 and 8 stars (Table S5).

A single study [24] was rated as “good” (8 stars) while quality of 4 studies [6,23,25,26] was considered “satisfactory (5–6 stars). Only one study received the lowest score (4 stars) [22]. The average quality score of the selected studies was 5.6 ± 1.3 stars.

## 4. Discussion

**Figure 4:**
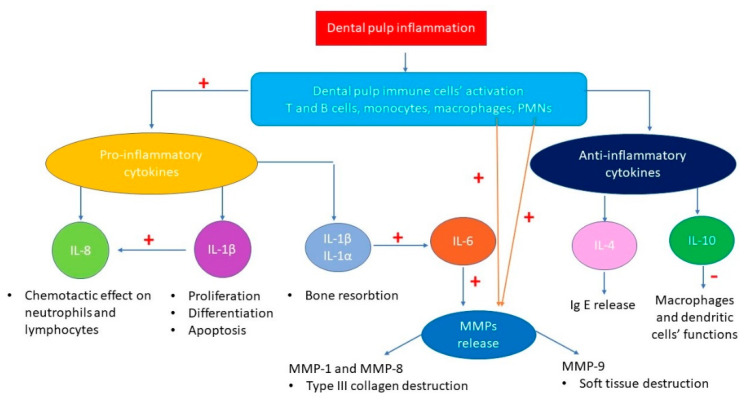
The ILs-MMPs interactions and their molecular consequences in the context of the dental pulp inflammation.

The studies selected in the present systematic review include ILs and/or MMPs that illustrated higher concentrations mostly in inflamed pulp tissue. O’Boskey et al. [15] showed that higher levels of ILs in the context of pulp inflammation can stimulate pulp cells to secrete MMPs, triggering extracellular matrix degradation in the pulp chamber (Figure 4).

ILs can have both inflammatory (IL-2, IL-6, IL-8) and anti-inflammatory (IL-4, IL-10, IL-13) effects, acting as sensitive inflammation modulators [24]. IL-1α and IL-1β have a key role in pro-inflammatory reactions as well as in bone resorption and cell proliferation via production of prostaglandin, cell-adhesion molecules and other pro-inflammatory cytokines such as IL-6 and IL-8 [27]. Levels of IL-1α analysed with multiplex assay illustrated a significant increase in both reversible (*p* < 0.01) and irreversible (*p* < 0.001) pulpitis groups compared to controls. In addition, IL-1α levels were significantly higher in irreversible inflamed pulps compared to reversible inflamed pulps, however without statistically significant differences [23].

IL-1β is an important mediator of essential cellular events, such as proliferation, differentiation and apoptosis. These cytokines levels were investigated using multiplex assay and showed a significant increase in both reversible (*p* < 0.01) and irreversible (*p* < 0.01) stages of dental pulp inflammation versus the control group. There were no significant differences between reversible and irreversible pulp inflammation [23]. Presence of IL-1β was also evaluated in chronically inflamed pulp tissue compared to healthy pulp using ELISA [6], with partial quite similar results with the previous study. IL-1β values were elevated in the chronic pulpitis group without statistical difference compared to healthy pulp [6]. Higher levels with statistical differences were observed in the group with chronic closed pulpitis both compared to the group with chronic opened pulpitis (*p* < 0.01) and to the control group (*p* < 0.01). On the other hand, in the same study, elevated levels of IL-1β were observed in healthy pulps compared to chronic open pulpitis, however without statistically significant differences [6], which may be due to the advanced inflammation degree in the pulp in open chronic pulpitis.

IL-4 promotes production of IgE and stimulation of mast cells [28]. Abd-Elmeguid et al. [23] showed that IL-1rα, IL-4 and IL-12 p40 are inflammatory mediators with a significant increase in the reversible and irreversible stages of dental pulp inflammation compared to normal pulp tissue, although with no significant differences between the two types of pulp inflammation. On the other hand, IL-7 and IL-13 levels were significantly higher in normal pulp tissue when compared to reversible and irreversible pulpitis. IL-15 levels were significantly elevated only in the reversible pulpitis group compared to controls (*p* < 0.01).

IL-6 has the potential to stimulate tissue degradation through the increase of MMPs levels and to induce the differentiation of mature B lymphocytes to plasma cells [29,30]. IL-8 acts as a chemotactic mediator for neutrophils and lymphocytes and is an interferon-c-inducing factor [27,31]. A study conducted in 2012 using the ELISA technique [22] showed significantly increased levels of IL-6 and IL-8 in the study group (*p* < 0.05), which consisted of inflamed dental pulp without differential diagnosis between reversible and irreversible inflammation. In accordance with the previous study, Dincer et al. have also shown elevated (*p* = 0.01) levels of IL-8 in irreversible inflamed pulps [24]. Similar results regarding IL-6 and IL-8 levels in reversible and irreversible inflamed pulp measured using ELISA have been obtained also using multiplex assay [23].

Extracellular matrix proteolysis is an essential event in the progression of inflammatory pathologies [32]. The presence of MMPs in inflamed and healthy pulp was also investigated, illustrating significantly higher levels in inflamed pulp tissue. MMP-1, MMP-8 and MMP-13 levels were assessed with ELISA. The obtained results reflected higher levels in chronic inflamed pulp versus healthy pulp tissue samples. These results may lead to the conclusion that these collagenases are involved in dental pulp collagen matrix degradation during chronic inflammation [25]. Sambandam et al. revealed that MMP-1 and MMP-8 are associated with the degradation of collagen type III [32]. Similar results regarding the significantly elevated (*p* < 0.001) levels of MMP-8 were observed in irreversibly inflamed pulp compared to normal pulp tissue, supporting its crucial role in inducing destruction of collagen in inflamed tissues [24].

In inflamed tissues, MMP-9 is secreted by PMNs and may represent the main reason for soft-tissue destruction in inflamed pulp [33]. MMP-9 or gelatinase B [34] can also be considered as a possible marker for pulpal inflammation, since its levels were significantly higher (*p* < 0.05) in inflamed primary teeth and inflamed permanent teeth than in normal teeth [26].

The quality assessment of the included studies using the modified Newcastle-Ottawa Quality Assessment scale [20,21] revealed weaknesses among the studies. That was observed by the fact that a single study was qualified as “good” [24]. Most frequent methodological flaws were observed in the representativeness of the sample as some studies included patients that seek treatment in university dental clinics [22,23,24,25] or they did not mention the provenience of the subjects [6,26], there was lack of information regarding *n* and/or the age of included subjects [6,22,23,26], there was no description of symptoms and tests for establishing the diagnosis of pulp inflammation [6,22,25] and also the possible confounding factors such as age, presence of systematic diseases or use of several medications were not taken into consideration [6,22,23,25,26]. On the other hand, each study was based on the same laboratory test in order to compare inflamed and normal pulp tissue levels of ILs and/or MMPs. All the studies included in this review clearly describe the statistical tests and the confidence intervals that were used.

The presence of studies with average quality, reflected by the mean score (5.6 ± 1.3 star) obtained after quality assessment may represent a limitation of the systematic review. Another important aspect is the distinction between the stages of inflammation, reversible and irreversible. As we mentioned above, two of the studies did not make a differentiation between these two types of afflictions and included both of them as a single experimental group [22,26]. A similar situation was observed in another study regarding chronic pulpitis without mentioning the type of chronic inflammation (open or closed) [25]. The differential diagnosis of these types of inflammation is a key feature, as the results of the included studies demonstrated. More precisely, the levels of some biomarkers are different in irreversible inflammation than those in reversible inflammation [6,23]. Despite this, we decided to include the mentioned studies because our focus was on the existence of an experimental group with pulp inflammation, as a consequence of dental caries’ progression, either reversible, irreversible or chronic.

Terminology regarding pulp inflammation varies and can lead to confusion and wrong diagnosis and treatment. Misleading terms and diagnoses exist for the same clinical condition as a result of the different classifications that mix clinical and histological terms [7]. A correct diagnosis depends on a detailed understanding of the pathology and also a precise knowledge of the diagnostic procedures. Another important aspect is the recognition of the healthy pulp tissue so that can be used as a comparative term regarding pulp pathology. In the present study we took into consideration all studies which include inflamed pulp tissue as a result of caries progression, either mentioned as reversible, irreversible or chronic inflammation.

In the future, evaluation of ILs and/or MMPs levels using molecular methods may represent a valuable tool for the clinician to establish the type and stage of pulp inflammation with precision. An accurate diagnosis will help in choosing the suitable treatment for each case. Overdiagnosis or underdiagnosis of the pulp condition followed by a wrong treatment may be associated with severe consequences. A reversible pulp inflammation diagnosis in a permanent immature tooth should be followed by a vital pulp therapy that must promote healing of the inflamed tissue and preservation of the healthy pulp’s vitality. An incorrect diagnosis of pulp pathology may lead to a total extraction of the dental pulp tissue, having a preserved future, as the root formation will be stopped and the thin dentinal walls will be susceptible to fracture. On the other hand, an incorrect diagnosis and undertreatment regarding irreversible pulpitis may lead to the progression of inflammation in the alveolar bone or even affect the general health.

## 5. Conclusions

In most of the studies included in the present systematic review, biological markers such as ILs and MMPs were mainly found in the experimental groups of inflamed pulp tissue having statistically significant differences compared to the healthy pulp samples. Although various laboratory tests were used, similar results were found between studies analysing the same biomarker. Biomarkers can help in studying various aspects of diseases such as inflammation and can be used as diagnostic tools. Patient symptoms and clinical findings are not always in concordance with the molecular diagnosis, which is more accurate. The study of molecular processes and evaluation of multiple parameters can be used in prevention, precise diagnosis or treatment of inflammatory diseases such as pulpitis. All of the biomarkers studied are promising markers to assess pulpal tissue health.

## Figures and Tables

**Table 1:** Studies included in the present systematic review analysing the presence of IL and MMPs in human dental pulp.

Reference	Clinical Diagnosis & *n* Per Group	Subjects (*n*) & Age (Mean Age ± SE)	Biomarker	Focus of the Study	Method	Comparison & Significance
Abd-Elmeguid et al., (2012) [22]	H (*n* = 5) INF (*n* = 12) (IF without differentiation) H (*n* = 8) INF (*n* = 20)	no data	IL-6	To examine the presence of dentin matrix protein-1 (DMP-1) in inflamed pulps; to define pulp inflammation in terms of cytokine expression.	ELISA	H/INF (R+IR): ↑ levels in INF; SS, *p* < 0.05
			IL-8			H/INF (R+IR): ↑ levels in INF; SS, *p* < 0.05
Abd-Elmeguid et al., (2013) [23]	H (*n* = 30) R INF (*n* = 23) IR INF (*n* = 12)	no data	IL-1α	To localize osteocalcin (OCN) in inflamed pulps, to distinguish its different levels in 2 stages of pulp inflammation and to suggest its possible interactions in pulpal inflammation; to identify the presence of different inflammatory and remodeling mediators in pulp tissue and measure their levels.	Multiplex Assay	H/R INF: ↑ levels in R INF; SS, *p* < 0.01 H/IR INF: ↑ levels in IR INF; SS, *p* < 0.001 R INF/IR INF: ↑ levels in IR INF but NS
			IL-1β			H/R INF: ↑ levels in R INF; SS, *p* < 0.01 H/IR INF: ↑ levels in IR INF; SS, *p* < 0.01 R INF/IR INF: ↑ levels in R INF but NS
			IL-1rα			H/R INF: ↑ levels in R INF; SS, *p* < 0.05 H/IR INF: ↑ levels in IR INF; SS, *p* < 0.05 R INF/IR INF: ↑ levels in IR INF but NS
			IL-4			H/R INF: ↑ levels in R INF; SS, *p* < 0.05 H/IR INF: ↑ levels in IR INF; SS, *p* < 0.05 R INF/IR INF: ↑ levels in IR INF but NS
			IL-6			H/R INF: ↑ levels in R INF; SS, *p* < 0.01 H/IR INF: ↑ levels in IR INF; SS, *p* < 0.05 R INF/IR INF: ↑ levels in R INF but NS
			IL-7			H/R INF: ↑ levels in H; SS, *p* < 0.001 H/IR INF: ↑ levels in H; SS, *p* < 0.01 R INF/IR INF: ↑ levels in R INF but NS
			IL-8			H/R INF: ↑ levels in H; SS, *p* < 0.05 H/IR INF: ↑ levels in H; SS, *p* < 0.01 R INF/IR INF: ↑ levels in R INF but NS
			IL-12 p40			H/R INF: ↑ levels in R INF; SS, *p* < 0.05 H/IR INF: ↑ levels in IR INF; SS, *p* < 0.01 R INF/IR INF: ↑ levels in IR INF but NS
			IL-13			H/R INF: ↑ levels in H; SS, *p* < 0.05 H/IR INF: ↑ levels in H; SS, *p* < 0.05 R INF/IR INF: ↑ levels in R INF but NS R INF/IR INF: ↑ levels in IR INF but NS
			IL-15			H/R INF: ↑ levels in R INF; SS, *p* < 0.01 H/IR INF: ↑ levels in IR INF but NS R INF/IR INF: ↑ levels in R INF but NS
Dincer at al., 2020 [24]	H (*n* = 20) IR INF (*n* = 20)	n = 40 16–50 yr. H:36.35 yr. IR INF: 22.50 yr.	IL-8	To compare the changes in amounts of NKA, SP, IL-8 and MMP-8 in pulp tissue	ELISA	H/IR INF: ↑ levels in IR INF; SS, *p* = 0/001
			MMP-8			H/IR INF: ↑ levels in IR INF; SS, *p* < 0.001
Evrosimovska et al., (2012) [25]	H (*n* = 10) CHR INF (*n* = 20)	n = 30 15–70 yr.	MMP-1 MMP-8 MMP-13	To determinate the concentration of MMP-1, -8 and -13 in the healthy pulp tissue of impacted third molars and to compare the values of the concentration between healthy and pathologically changed tissue	ELISA	H/CHR INF: ↑ levels in CHR INF; SS, *p* < 0.01
Subaric et al., (2017) [6]	H (*n* = 12) CHR AP INF (*n* = 22) CHR CL INF (*n* = 19)	no data	IL-1β	To determine the IL-1β concentrations in chronically inflamed and healthy dental pulp	ELISA	H/CHR INF (AP+CL): ↑ levels in CHR INF but NS, *p* = 0.590 H/CHR CL INF/CHR AP INF: ↑ levels in CHR CL INF; SS, *p* < 0.01 H/CHR CL INF: ↑ levels in CHR CL INF; SS, *p* < 0.01 H/CHR AP INF: ↑ levels in H but NS, *p* = 0.081 CHR CL INF/CHR AP INF: ↑ levels in CHR CL INF; SS, *p* < 0.01
Suwanchai et al., (2011) [26]	H(P) (*n* = 18) H (T) (*n* = 7) IR INF (P) (*n* = 7) R INF (T) (*n* = 8) IR INF (T) (*n* = 8)	*n* = no data H(P): 17.3 ± 1.1 yr. IR INF(P): 35.4 ± 6.3 yr. H(T): 9.4 ± 0.9 yr. R + IR INF(T): 6.1 ± 0.7 yr.	MMP-9	To investigate alterations in Na_v_1.8 and Na_v_1.9 expression within inflamed dental pulp tissue of human primary teeth	Western Blot	H(P)/IR INF(P): ↑ levels in IR INF; SS, *p* < 0.05 H(T)/R+IR INF (without differentiation): ↑ levels in R+IR INF; SS, *p* < 0.05

SS = statistically significant difference; NS = no statistical difference. H = healthy pulp; R INF = reversible inflammation; IR INF = irreversible inflammation; CHR INF = chronic inflammation; CHR AP INF = chronic open inflammation (chronica aperta pulpitis); CHR CL INF = chronic closed inflammation (chronica clausa pulpitis); P = permanent tooth; T = temporary tooth; yr = years.

## Data Availability

Not applicable.

## Supplementary Materials

The following are available online, Table S1: Search strategies employed for this literature review, Table S2: Hits from the literature search obtained with the different databases, Table S3: Reports not retrieved, Table S4: Studies excluded with reasons, Table S5: Newcastle-Ottawa Scale adapted for cross-sectional studies used for quality assessment of the present systematic review, Table S6: PRISMA checklist, Table S7: PRISMA abstract checklist.

## Sample Availability

Samples of the compounds not applicable.
