# Supplementary material for: ILs and MMPs Levels in Inflamed Human Dental Pulp: A Systematic Review
Source: Molecules. 2021 Jul 7;26(14):4129. doi: 10.3390/molecules26144129 (PMC8304045; doi:10.3390/molecules26144129)
Supplement: Supplementary file 1 [file molecules-26-04129-s001.zip › molecules-1270516-supplementary.pdf]

SUPPLEMENTARY MATERIAL

Table S1: Search strategies employed for this literature review

**Database**	OVID
**Date**	May 4, 2021
**Search**	1 interleukins/ or interleukin-1/ or interleukin-2/ or interleukin-3/ or interleukin-4/ or interleukin-5/ or interleukin-6/ or interleukin-7/ or interleukin-8/ or interleukin-9/ or interleukin-10/ or interleukin-11/ or interleukin-12/ or interleukin-13/ or interleukin-15/ or interleukin-16/ or interleukin-17/ or interleukin-18/ or interleukin-23/ or interleukin- 27/ or interleukin-33/ (230422)<br>2 interleukins.ti,ab,kw. (6982)<br>3 1 or 2 (234355)<br>4 Pulpitis/ (2833)<br>5 dental pulp inflammation.mp. (69)<br>6 pulpitis.ti,ab,kw. (1757)<br>7 4 or 5 or 6 (3553)<br>8 3 and 7 (80)<br>9 Matrix Metalloproteinases/ (10153)<br>10 matrix metalloproteinases.ti,ab,kw. (20914)<br>11 9 or 10 (25577)<br>12 7 and 11 (17)<br>13 8 or 12 (93)
**Total articles retrieved**	93

Table S1: *(continued)*

**Database**	Web of Science
**Date**	May 5, 2021
**Search**	#9 (56) #8 OR #7 OR #5 OR #3<br>#8 (38) #6 AND #4<br>#7 (15) #6 AND #2<br>#6 (43 559) TS=(matrix metalloproteinases)<br>#5 (9) #4 AND #1<br>#4 (1 217) TS=(dental pulp inflammation)<br>#3 (3) #2 AND #1<br>#2 (1 327) TS=(pulpitis)<br>#1 (7 941) TS=(interleukins)
**Total articles retrieved**	56

Table S1: *(continued)*

**Database**	PubMed
**Date**	May 5, 2021
**Search**	(("interleukine"[All Fields] OR "interleukines"[All Fields] OR "interleukins"[MeSH Terms] OR "interleukins"[All Fields] OR "interleukin"[All Fields]) AND ("pulpitis"[MeSH Terms] OR "pulpitis"[All Fields] OR "pulpitides"[All Fields])) OR (("interleukine"[All Fields] OR "interleukines"[All Fields] OR "interleukins"[MeSH Terms] OR "interleukins"[All Fields] OR "interleukin"[All Fields]) AND "dental pulp inflammation"[All Fields]) OR ("matrix metalloproteinases"[All Fields] AND ("pulpitis"[MeSH Terms] OR "pulpitis"[All Fields] OR "pulpitides"[All Fields])) OR ("matrix metalloproteinases"[All Fields] AND "dental pulp inflammation"[All Fields])
**Total articles retrieved**	136

Table S1: *(continued)*

**Database**	Wiley Online Library
**Date**	May 5, 2021
**Search**	[[All: interleukins] OR [All: il] OR [All: "matrix metalloproteinases"] OR [All: mmp]] AND [[All: pulpitis] OR [All: "dental pulp inflammation"]]
**Total articles retrieved**	369

Table S1: *(continued)*

**Database**	SCOPUS
**Date**	May 8, 2021
**Search**	TITLE-ABS-KEY ((interleukins AND pulpitis)) OR (interleukins AN D "dental pulp inflammation") OR ("matrix metalloproteinases" AN D pulpitis) OR ("matrix metalloproteinases" AND "dental pulp inflam mation")
**Total articles retrieved**	372

Table S2: Hits from the literature search obtained with the different databases

Database	Inception of database	Hits	After duplicate removal
PubMed	1995	136	14
Ovid	1996	93	17
Scopus	1990	372	287
Web of Science	1998	56	43
Wiley Online Library	1984	369	358
**TOTAL**		**1026**	**714**
Additional records identified through other sources	0	0	0

Table S3: Reports not retrieved

Reference	Report
[1]	1. 5-aza-2’-deoxycytidine may regulate the inflammatory response of human odontoblast-like cells through the NF-κB pathway
[2]	2. Detection of interleukin-8 in exudates from normal and inflamed human dental pulp tissues.
[3]	3. The role of cytokines in pulp inflammation
[4]	4. Long noncoding RNA MEG3 expressed in human dental pulp regulates LPS-Induced inflammation and odontogenic differentiation in pulpitis
[5]	5. NUTM2A-AS1 silencing alleviates LPS-induced apoptosis and inflammation in dental pulp cells through targeting let-7c-5p/HMGB1 axis.
[6]	6. NLRP6-caspase 4 inflammasome activation in response to cariogenic bacterial lipoteichoic acid in human dental pulp inflammation.

Table S4: Studies excluded with reasons

**Animal studies/studies not on human dental pulp**
Ayre W.N, 2018[7]; Bletsa A, 2006[8]; Bletsa A, 2009[9]; Eba H, 2012[10]; Takimoto K, 2014[11]; Tani-Ishii N, 1995[12]; Wang J, 2019[13]; Yuan H, 2018[14]; Zheng L, 2009[15]
**Studies on stem cell/cell cultures only**
Adachi T, 2007[16]; Al-Sharabi N., 2017 [17]; Barkhordar R.A, 2002[18]; Bei Y., 2016[19]; Bindal P, 2018[20]; Cai L., 2020[21]; Carrouel F., 2013[22]; Chang Y.C, 2001[23]; Chang Y.C, 2003[24]; Chang M.C, 2006[25]; Chang M.C, 2009[26]; Chang M.C, 2012[27]; Dommisch H., 2007[28]; Du W., 2012 [29]; Engels-Deutch M, 2003[30]; Feng Z, 2019[31]; Goda S, 2015[32]; He J, 2021[33]; He W, 2013[34]; Lee S, 2016[35]; Lee Y.Y, 2011[36]; Lin S.K, 2001 [37]Lin S.K, 2002[38]; Lu H.X, 2002[39]; Luo H, 2018[40]; Mahmoudi J, 2017[41]; Meng R, 2019[42]; Min K, 2008[43]; Park S, 2004[44]; Pereira L, 2012[45]; Rhim E.M, 2013[46]; Satrawaha S, 2011[47]; Shindo S, 2021[48]; Soares D. G, 2018[49]; Song F, 2017[50]; Sugiuchi A, 2018[51]; Takanche J.S, 2018[52]; Takegawa D, 2014[53]; Tamura M, 1996[54]; Teja K.V, 2018[55]; Tjaderhane L, 2002[56]; Tokuda M, 2001[57]; Tsai A.I, 2017[58]; Wang D, 2021[59]; Wang F, 2020[60]; Wang X, 2018[61]; Wei L, 2018[62]; Widbiller M, 2018[63]; Wisithphrom K, 2006[64]; Xue D, 2018[65]; Yamada Y, 2019[66]; Yang G, 2019[67]; Yang L.C, 2003[68]; Yonehiro J, 2012[69]; Yu M.K, 2009[70]; Yu S, 2014[71]; Zhai S, 2013[72]; Zhai Y, 2019[73]; Zhai Y, 2020[74]; Zhao Y, 2014[75]
**No potential biomarker was investigated**
Lei F, 2019[76]
**Only histological findings, presence of cells, bacteria, viruses**
Bruno K.F, 2010[77]; Giorgini E, 2017[78]
**Review articles**
Anshida V.P, 2020[79], Arora S., 2021[80]; Emilia E, 2015[81]; Hirsch V, 2017[82]; Khorasani M.M.Y, 2020[83]; Nibali L, 2012[84]; Rechenberg D.K, 2016[85]; Sambandam V, 2014[86]; Zanini M, 2017[87]

Table S4: (*continued)*

**Studies using other methods to quantify IL and/or MMPs than those specified**
Accorsi-Mendonça T, 2013[88]; Alvarez M.M.P, 2017[89]; Brodzikowska A, 2019[90]; Galicia J.C, 2016[91]; Gatta V, 2012[92]; Liu M, 2019[93]; Tsai C.H, 2005[94]; Xiong H, 2015[95]; Zehnder M, 2003[96]; Zhang N, 2019[97]
**Studies analysing other substrate than that chosen to test**
Aishuwariya T, 2021[98]; Ballal V, 2018[99]; Brizuela C, 2020[100]; ElSalhy M, 2013[101]; Mente J, 2016[102]; Nakanishi T, 1995[103]; Rechenberg D.K, 2014[104]; Sharma R, 2021[105]; Shimada Y, 2009[106]; Zehnder M, 2011[107]; Zhu Z.Y, 2012[108]
**Control group not existing/other than normal pulp tissue**
Wahlgren J, 2002[109]; Zhou S, 2019[110]
**Studies that took place and published before 2011**
Anderson L.M, 2002[111]; Barkhordar R.A, 1999[112]; Gusman H, 2002[113]; Huang G.T, 1999[114]; Rauschenberger C.R, 1997[115]; Sattari M, 2009[116]; Shin S.J, 2002[117]; Silva A.C, 2009[118]

Table S5: Newcastle-Ottawa Scale adapted for cross-sectional studies used for quality assessment of the present systematic review

NOS criteria	Abd-Elmeguid et al. (2012)[119]	Abd-Elmeguid et al. (2013)[120]	Dincer et al. (2020)[121]	Evrosimovska et al. (2012)[122]	Subaric et al. (2011)[123]	Suwanchai et al. (2011)[124]
**Selection**						
1. Representativeness of the sample	-	-	-	-	-	-
2. Sample size	*	*	*	*	*	*
3. Non-responders	-	*	*	-	_	*
4. Ascertainment of the exposure (risk factor)	-	*	*	*	*	*
**Comparability**						
1. The study controls for the most important factor	-	-	*	-	-	-
2. The study controls for any additional factor	-	-	*	-	-	-
**Outcome**						
1. Assessment of outcome	**	**	**	**	**	**
2. Statistical test	*	*	*	*	*	*
**Total score**	4	6	8	5	5	6
	unsatisfactory	satisfactory	Good	satisfactory	satisfactory	satisfactory

Selection: (Maximum 5 stars)

1) Representativeness of the sample:
   a) Truly representative of the average in the target population. * (all subjects or random sampling)
   b) Somewhat representative of the average in the target population. * (non-random sampling)
   c) Selected group of users.
   d) No description of the sampling strategy.

2) Sample size:
   a) Justified and satisfactory. *
   b) Not justified.

3) Non-respondents:
   a) Comparability between respondents and non-respondents characteristics is established, and the response rate is satisfactory. *
   b) The response rate is unsatisfactory, or the comparability between respondents and non-respondents is unsatisfactory.
   c) No description of the response rate or the characteristics of the responders and the non-responders.

4) Ascertainment of the exposure (risk factor):
   a) Validated measurement tool (clinical diagnosis confirmed histologically) **
   b) Non-validated measurement tool (clinical diagnosis), but the tool is available or described.*
   c) No description of the measurement tool.

Comparability: (Maximum 2 stars)

1) The subjects in different outcome groups are comparable, based on the study design or analysis. Confounding factors are controlled .*
   a) The study controls for the most important factor (select one). *
   b) The study control for any additional factor. *

*most important factor is different for each study; age, presence of systemic diseases, medications, use of rx for diagnosis establishment

Outcome: (Maximum 3 stars)

1) Assessment of the outcome:

a) Independent blind assessment, or confirmation of the outcome by reference tests (ELISA, Multiplex assay, Western blot) **

b) Record linkage. **

c) Self report. *

d) No description.

2) Statistical test:

a) The statistical test used to analyze the data is clearly described and appropriate, and the measurement of the association is presented, including confidence intervals and the probability level (p value). *

b) The statistical test is not appropriate, not described, or incomplete.

Table S6: PRISMA checklist

Section and Topic	Item #	Checklist item	Location where item is reported
**TITLE**			
Title	1	Identify the report as a systematic review.	p.1
**ABSTRACT**			
Abstract	2	See the PRISMA 2020 for Abstracts checklist.	p.1
**INTRODUCTION**			
Rationale	3	Describe the rationale for the review in the context of existing knowledge.	p.2-4
Objectives	4	Provide an explicit statement of the objective(s) or question(s) the review addresses.	p.3,4
**METHODS**			
Eligibility criteria	5	Specify the inclusion and exclusion criteria for the review and how studies were grouped for the syntheses.	p.4
Information sources	6	Specify all databases, registers, websites, organisations, reference lists and other sources searched or consulted to identify studies. Specify the date when each source was last searched or consulted.	p.4, Table S2
Search strategy	7	Present the full search strategies for all databases, registers and websites, including any filters and limits used.	Table S1
Selection process	8	Specify the methods used to decide whether a study met the inclusion criteria of the review, including how many reviewers screened each record and each report retrieved, whether they worked independently, and if applicable, details of automation tools used in the process.	p.4, 6
Data collection process	9	Specify the methods used to collect data from reports, including how many reviewers collected data from each report, whether they worked independently, any processes for obtaining or confirming data from study investigators, and if applicable, details of automation tools used in the process.	p.6
Data items	10a	List and define all outcomes for which data were sought. Specify whether all results that were compatible with each outcome domain in each study were sought (e.g. for all measures, time points, analyses), and if not, the methods used to decide which results to collect.	p.6, Table 1
	10b	List and define all other variables for which data were sought (e.g. participant and intervention characteristics, funding sources). Describe any assumptions made about any missing or unclear information.	p.6, Table 1
Study risk of bias assessment	11	Specify the methods used to assess risk of bias in the included studies, including details of the tool(s) used, how many reviewers assessed each study and whether they worked independently, and if applicable, details of automation tools used in the process.	p.6, Table S5
Effect measures	12	Specify for each outcome the effect measure(s) (e.g. risk ratio, mean difference) used in the synthesis or presentation of results.	Table 1
Synthesis methods	13a	Describe the processes used to decide which studies were eligible for each synthesis (e.g. tabulating the study intervention characteristics and comparing against the planned groups for each synthesis (item #5)).	p.4-6
	13b	Describe any methods required to prepare the data for presentation or synthesis, such as handling of missing summary statistics, or data conversions.	NA
	13c	Describe any methods used to tabulate or visually display results of individual studies and syntheses.	p.6, Table 1

Table S6: *(continued)*

	13d	Describe any methods used to synthesize results and provide a rationale for the choice(s). If meta-analysis was performed, describe the model(s), method(s) to identify the presence and extent of statistical heterogeneity, and software package(s) used.	NA
	13e	Describe any methods used to explore possible causes of heterogeneity among study results (e.g. subgroup analysis, meta-regression).	NA
	13f	Describe any sensitivity analyses conducted to assess robustness of the synthesized results.	NA
Reporting bias assessment	14	Describe any methods used to assess risk of bias due to missing results in a synthesis (arising from reporting biases).	NA
Certainty assessment	15	Describe any methods used to assess certainty (or confidence) in the body of evidence for an outcome.	NA
**RESULTS**			
Study selection	16a	Describe the results of the search and selection process, from the number of records identified in the search to the number of studies included in the review, ideally using a flow diagram.	p.4,6<br>Figure 2
	16b	Cite studies that might appear to meet the inclusion criteria, but which were excluded, and explain why they were excluded.	Table S4
Study characteristics	17	Cite each included study and present its characteristics.	Table 1
Risk of bias in studies	18	Present assessments of risk of bias for each included study.	p.12,<br>Table S5
Results of individual studies	19	For all outcomes, present, for each study: (a) summary statistics for each group (where appropriate) and (b) an effect estimate and its precision (e.g. confidence/credible interval), ideally using structured tables or plots.	Table 1
Results of syntheses	20a	For each synthesis, briefly summarise the characteristics and risk of bias among contributing studies.	p.12
	20b	Present results of all statistical syntheses conducted. If meta-analysis was done, present for each the summary estimate and its precision (e.g. confidence/credible interval) and measures of statistical heterogeneity. If comparing groups, describe the direction of the effect.	p.11,12
	20c	Present results of all investigations of possible causes of heterogeneity among study results.	NA
	20d	Present results of all sensitivity analyses conducted to assess the robustness of the synthesized results.	NA
Reporting biases	21	Present assessments of risk of bias due to missing results (arising from reporting biases) for each synthesis assessed.	NA
Certainty of evidence	22	Present assessments of certainty (or confidence) in the body of evidence for each outcome assessed.	NA
**DISCUSSION**			
Discussion	23a	Provide a general interpretation of the results in the context of other evidence.	p.12-15
	23b	Discuss any limitations of the evidence included in the review.	p.14
	23c	Discuss any limitations of the review processes used.	p.14
	23d	Discuss implications of the results for practice, policy, and future research.	p.14

Table S6: *(continued)*

OTHER INFORMATION			
Registration and protocol	24a	Provide registration information for the review, including register name and registration number, or state that the review was not registered.	NA
	24b	Indicate where the review protocol can be accessed, or state that a protocol was not prepared.	NA
	24c	Describe and explain any amendments to information provided at registration or in the protocol.	NA
Support	25	Describe sources of financial or non-financial support for the review, and the role of the funders or sponsors in the review.	p.15
Competing interests	26	Declare any competing interests of review authors.	p.15
Availability of data, code and other materials	27	Report which of the following are publicly available and where they can be found: template data collection forms; data extracted from included studies; data used for all analyses; analytic code; any other materials used in the review.	p.15

From: Page MJ, McKenzie JE, Bossuyt PM, Boutron I, Hoffmann TC, Mulrow CD, et al. The PRISMA 2020 statement: an updated guideline for reporting systematic reviews. BMJ 2021;372:n71. doi: 10.1136/bmj.n71 [125]

Table S7: PRISMA abstract checklist

Section and Topic	Item #	Checklist item	Reported (Yes/No)
**TITLE**			
Title	1	Identify the report as a systematic review.	Yes
**BACKGROUND**			
Objectives	2	Provide an explicit statement of the main objective(s) or question(s) the review addresses.	Yes
**METHODS**			
Eligibility criteria	3	Specify the inclusion and exclusion criteria for the review.	No
Information sources	4	Specify the information sources (e.g. databases, registers) used to identify studies and the date when each was last searched.	Yes partially
Risk of bias	5	Specify the methods used to assess risk of bias in the included studies.	Yes
Synthesis of results	6	Specify the methods used to present and synthesise results.	No
**RESULTS**			
Included studies	7	Give the total number of included studies and participants and summarise relevant characteristics of studies.	Yes
Synthesis of results	8	Present results for main outcomes, preferably indicating the number of included studies and participants for each. If meta-analysis was done, report the summary estimate and confidence/credible interval. If comparing groups, indicate the direction of the effect (i.e. which group is favoured).	Yes partially
**DISCUSSION**			
Limitations of evidence	9	Provide a brief summary of the limitations of the evidence included in the review (e.g. study risk of bias, inconsistency and imprecision).	No
Interpretation	10	Provide a general interpretation of the results and important implications.	Yes
**OTHER**			
Funding	11	Specify the primary source of funding for the review.	No
Registration	12	Provide the register name and registration number.	No

From: Page MJ, McKenzie JE, Bossuyt PM, Boutron I, Hoffmann TC, Mulrow CD, et al. The PRISMA 2020 statement: an updated guideline for reporting systematic reviews. BMJ 2021;372:n71. doi: 10.1136/bmj.n71 [125]

References

1. Feng, Z.; Meng, R.; Li, Q.; Li, D.; Xu, Q. 5-aza-2'-deoxycytidine may regulate the inflammatory response of human odontoblast-like cells through the NF-κB pathway. *n/a*, doi:https://doi.org/10.1111/iej.13488.
2. Guo, X.; Niu, Z.; Xiao, M.; Yue, L.; Lu, H. Detection of interleukin-8 in exudates from normal and inflamed human dental pulp tissues. *The Chinese journal of dental research : the official journal of the Scientific Section of the Chinese Stomatological Association (CSA)* **2000**, *3*, 63-66.
3. Kokkas, A.; Goulas, A.; Stavrianos, C.; Anogianakis, G. The role of cytokines in pulp inflammation. *Journal of biological regulators and homeostatic agents* **2011**, *25*, 303-311.
4. Liu, M.; Chen, L.; Wu, J.; Lin, Z.; Huang, S. Long noncoding RNA MEG3 expressed in human dental pulp regulates LPS-Induced inflammation and odontogenic differentiation in pulpitis. *Experimental cell research* **2021**, *400*, doi:10.1016/j.yexcr.2021.112495.
5. Wang, X.; Sun, H.; Hu, Z.; Mei, P.; Wu, Y.; Zhu, M. NUTM2A-AS1 silencing alleviates LPS-induced apoptosis and inflammation in dental pulp cells through targeting let-7c-5p/HMGB1 axis. *International immunopharmacology* **2021**, *96*, doi:10.1016/j.intimp.2021.107497.
6. Tian, X.X.; Li, R.; Liu, C.; Liu, F.; Yang, L.J.; Wang, S.P.; Wang, C.L. NLRP6-caspase 4 inflammasome activation in response to cariogenic bacterial lipoteichoic acid in human dental pulp inflammation. *International endodontic journal* **2020**, doi:10.1111/iej.13469.
7. Ayre, W.N.; Melling, G.; Cuveillier, C.; Natarajan, M.; Roberts, J.L.; Marsh, L.L.; Lynch, C.D.; Maillard, J.Y.; Denyer, S.P.; Sloan, A.J. Enterococcus faecalis demonstrates pathogenicity through increased attachment in an ex vivo polymicrobial pulpal infection. *Infection and immunity* **2018**, *86*, doi:10.1128/IAI.00871-17.
8. Bletsa, A.; Berggreen, E.; Fristad, I.; Tenstad, O.; Wiig, H. Cytokine signalling in rat pulp interstitial fluid and transcapillary fluid exchange during lipopolysaccharide-induced acute inflammation. **2006**, *573*, 225-236, doi:https://doi.org/10.1113/jphysiol.2006.104711.
9. Bletsa, A.; Fristad, I.; Berggreen, E. Sensory pulpal nerve fibres and trigeminal ganglion neurons express IL-1RI: a potential mechanism for development of inflammatory hyperalgesia. **2009**, *42*, 978-986, doi:https://doi.org/10.1111/j.1365-2591.2009.01605.x.
10. Eba, H.; Murasawa, Y.; Iohara, K.; Isogai, Z.; Nakamura, H.; Nakamura, H.; Nakashima, M. The Anti-Inflammatory Effects of Matrix Metalloproteinase-3 on Irreversible Pulpitis of Mature Erupted Teeth. *PloS one* **2012**, *7*, doi:10.1371/journal.pone.0052523.
11. Takimoto, K.; Kawashima, N.; Suzuki, N.; Koizumi, Y.; Yamamoto, M.; Nakashima, M.; Suda, H. Down-regulation of Inflammatory Mediator Synthesis and Infiltration of Inflammatory Cells by MMP-3 in Experimentally Induced Rat Pulpitis. *Journal of Endodontics* **2014**, *40*, 1404-1409, doi:10.1016/j.joen.2014.04.001.
12. Tani-Ishii, N.; Wang, C.-Y.; Stashenko, P. Immunolocalization of bone-resorptive cytokines in rat pulp and periapical lesions following surgical pulp exposure. **1995**, *10*, 213-219, doi:https://doi.org/10.1111/j.1399-302X.1995.tb00145.x.
13. Wang, J.; Du, Y.; Deng, J.; Wang, X.; Long, F.; He, J. MicroRNA-506 is involved in regulation of the occurrence of lipopolysaccharides (LPS)-induced pulpitis by sirtuin 1 (SIRT1). *Medical Science Monitor* **2019**, *25*, 10008-10015, doi:10.12659/MSM.918172.

14. Yuan, H.; Zhang, H.; Hong, L.; Zhao, H.; Wang, J.; Li, H.; Che, H.; Zhang, Z. MicroRNA let-7c-5p suppressed lipopolysaccharide-induced dental pulp inflammation by inhibiting dentin matrix protein-1-mediated nuclear factor kappa b (NF-κb) pathway in vitro and in vivo. *Medical Science Monitor* **2018**, *24*, 6656-6665, doi:10.12659/MSM.909093.
15. Zheng, L.; Amano, K.; Iohara, K.; Ito, M.; Imabayashi, K.; Into, T.; Matsushita, K.; Nakamura, H.; Nakashima, M. Matrix Metalloproteinase-3 Accelerates Wound Healing following Dental Pulp Injury. *American Journal of Pathology* **2009**, *175*, 1905-1914, doi:10.2353/ajpath.2009.080705.
16. Adachi, T.; Nakanishi, T.; Yumoto, H.; Hirao, K.; Takahashi, K.; Mukai, K.; Nakae, H.; Matsuo, T. Caries-related bacteria and cytokines induce CXCL10 in dental pulp. *Journal of dental research* **2007**, *86*, 1217-1222, doi:10.1177/154405910708601215.
17. Al-Sharabi, N.; Mustafa, M.; Ueda, M.; Xue, Y.; Mustafa, K.; Fristad, I. Conditioned medium from human bone marrow stromal cells attenuates initial inflammatory reactions in dental pulp tissue. **2017**, *33*, 19-26, doi:https://doi.org/10.1111/edt.12277.
18. Barkhordar, R.A.; Ghani, Q.P.; Russell, T.R.; Hussain, M.Z. Interleukin-1beta activity and collagen synthesis in human dental pulp fibroblasts. *Journal of Endodontics* **2002**, *28*, 157-159.
19. Bei, Y.; Tianqian, H.; Fanyuan, Y.; Haiyun, L.; Xueyang, L.; Jing, Y.; Chenglin, W.; Ling, Y. ASH1L Suppresses Matrix Metalloproteinase through Mitogen-activated Protein Kinase Signaling Pathway in Pulpitis. *Journal of Endodontics* **2017**, *43*, 306-314.e302, doi:10.1016/j.joen.2016.10.020.
20. Bindal, P.; Ramasamy, T.S.; Abu Kasim, N.H.; Gnanasegaran, N.; Chai, W.L. Immune responses of human dental pulp stem cells in lipopolysaccharide-induced microenvironment. *Cell biology international* **2018**, *42*, 832-840, doi:10.1002/cbin.10938.
21. Cai, L.; Zhan, M.; Li, Q.; Li, D.; Xu, Q. DNA methyltransferase DNMT1 inhibits lipopolysaccharide-induced inflammatory response in human dental pulp cells involving the methylation changes of IL-6 and TRAF6. *Molecular medicine reports* **2020**, *21*, 959-968, doi:10.3892/mmr.2019.10860.
22. Carrouel, F.; Staquet, M.J.; Keller, J.F.; Baudouin, C.; Msika, P.; Bleicher, F.; Alliot-Licht, B.; Farges, J.C. Lipopolysaccharide-binding protein inhibits toll-like receptor 2 activation by lipoteichoic acid in human odontoblast-like cells. *Journal of Endodontics* **2013**, *39*, 1008-1014, doi:10.1016/j.joen.2013.04.020.
23. Chang, Y.C.; Yang, S.F.; Hsieh, Y.S. Regulation of matrix metalloproteinase-2 production by cytokines and pharmacological agents in human pulp cell cultures. *Journal of Endodontics* **2001**, *27*, 679-682, doi:10.1097/00004770-200111000-00007.
24. Chang, Y.C.; Yang, S.F.; Huang, F.M.; Liu, C.M.; Tai, K.W.; Hsieh, Y.S. Proinflammatory cytokines induce cyclooxygenase-2 mRNA and protein expression in human pulp cell cultures. *Journal of Endodontics* **2003**, *29*, 201-204, doi:10.1097/00004770-200303000-00009.
25. Chang, M.C.; Chen, Y.J.; Tai, T.F.; Tai, M.R.; Li, M.Y.; Tsai, Y.L.; Lan, W.H.; Wang, Y.L.; Jeng, J.H. Cytokine-induced prostaglandin e2 production and cyclooxygenase-2 expression in dental pulp cells: Downstream calcium signalling via activation of prostaglandin EP receptor. *International endodontic journal* **2006**, *39*, 819-826, doi:10.1111/j.1365-2591.2006.01156.x.

26. Chang, M.C.; Chang, H.H.; Lee, M.Y.; Lin, C.C.; Yeh, H.W.; Yang, T.T.; Lin, P.S.; Tseng, W.Y.; Jeng, J.H. Prostaglandin F2α-Induced Interleukin-8 Production in Human Dental Pulp Cells Is Associated With MEK/ERK Signaling. *Journal of Endodontics* **2009**, *35*, 508-512, doi:10.1016/j.joen.2008.12.023.
27. Chang, M.C.; Lin, L.D.; Zwei-Ching Chang, J.; Huang, C.F.; Chuang, F.H.; Lee, J.J.; Jeng, P.Y.; Wang, T.M.; Jeng, J.H. Regulation of vascular cell adhesion molecule-1 in dental pulp cells by interleukin-1β: The role of prostanoids. *Journal of Endodontics* **2012**, *38*, 774-779, doi:10.1016/j.joen.2012.02.030.
28. Dommisch, H.; Winter, J.; Willebrand, C.; Eberhard, J.; Jepsen, S. Immune regulatory functions of human beta-defensin-2 in odontoblast-like cells. **2007**, *40*, 300-307, doi:https://doi.org/10.1111/j.0143-2885.2007.01228.x.
29. Du, W.; Jia, M. Effect of lipopolysaccharide and transforming growth factor-beta 1 on expression and signal pathway of Toll like receptor 4 in dental pulp cells. *Hua xi kou qiang yi xue za zhi = Huaxi kouqiang yixue zazhi = West China journal of stomatology* **2012**, *30*, 77-"81, 86".
30. Engels-Deutsch, M.; Pini, A.; Yamashita, Y.; Shibata, Y.; Haikel, Y.; Schöller-Guinard, M.; Klein, J.P. Insertional inactivation of pac and rmlB genes reduces the release of tumor necrosis factor alpha, interleukin-6, and interleukin-8 induced by Streptococcus mutans in monocytic, dental pulp, and periodontal ligament cells. *Infection and immunity* **2003**, *71*, 5169-5177, doi:10.1128/IAI.71.9.5169-5177.2003.
31. Feng, Z.; Zhan, M.; Meng, R.; Wang, X.; Xu, Q. 5-Aza-2'-deoxycytidine enhances lipopolysaccharide-induced inflammatory cytokine expression in human dental pulp cells by regulating TRAF6 methylation. *Bioengineered* **2019**, *10*, 197-206, doi:10.1080/21655979.2019.1621135.
32. Goda, S.; Kato, Y.; Domae, E.; Hayashi, H.; Tani-Ishii, N.; Iida, J.; Ikeo, T. Effects of JNK1/2 on the inflammation cytokine TNF-α-enhanced production of MMP-3 in human dental pulp fibroblast-like cells. **2015**, *48*, 1122-1128, doi:https://doi.org/10.1111/iej.12411.
33. He, J.; Qin, M.; Chen, Y.; Hu, Z.; Ye, L.; Hui, T. EZH2 Promotes Extracellular Matrix Degradation via Nuclear Factor-κB (NF-κB) and p38 Signaling Pathways in Pulpitis. *Inflammation* **2021**, doi:10.1007/s10753-021-01470-7.
34. He, W.; Qu, T.; Yu, Q.; Wang, Z.; Lv, H.; Zhang, J.; Zhao, X.; Wang, P. LPS induces IL-8 expression through TLR4, MyD88, NF-kappaB and MAPK pathways in human dental pulp stem cells. **2013**, *46*, 128-136, doi:https://doi.org/10.1111/j.1365-2591.2012.02096.x.
35. Lee, S.; Zhang, Q.Z.; Karabucak, B.; Le, A.D. DPSCs from inflamed pulp modulate macrophage function via the TNF-α/IDO axis. *Journal of dental research* **2016**, *95*, 1274-1281, doi:10.1177/0022034516657817.
36. Lee, Y.Y.; Chan, C.H.; Hung, S.L.; Chen, Y.C.; Lee, Y.H.; Yang, S.F. Up-regulation of nucleotide-binding oligomerization domain 1 in inflamed human dental pulp. *Journal of Endodontics* **2011**, *37*, 1370-1375, doi:10.1016/j.joen.2011.06.008.
37. Lin, S.K.; Wang, C.C.; Huang, S.; Lee, J.J.; Chiang, C.P.; Lan, W.H.; Hong, C.Y. Induction of dental pulp fibroblast matrix metalloproteinase-1 and tissue inhibitor of metalloproteinase-1 gene expression by interleukin-1alpha and tumor necrosis factor-alpha through a prostaglandin-dependent pathway. *J Endod* **2001**, *27*, 185-189, doi:10.1097/00004770-200103000-00012.

38. Lin, S.K.; Kuo, M.Y.; Wang, J.S.; Lee, J.J.; Wang, C.C.; Huang, S.; Shun, C.T.; Hong, C.Y. Differential regulation of interleukin-6 and inducible cyclooxygenase gene expression by cytokines through prostaglandin-dependent and -independent mechanisms in human dental pulp fibroblasts. *J Endod* **2002**, *28*, 197-201, doi:10.1097/00004770-200203000-00013.
39. Lu, H.X.; Xiao, M.Z.; Niu, Z.Y.; Guo, X.M.; Zhao, S.L.; Wang, H.G.; Guo, H.Y. Effect of IL-1ra on human dental pulp cells and pulpal inflammation. *International endodontic journal* **2002**, *35*, 807-811, doi:10.1046/j.1365-2591.2002.00542.x.
40. Luo, H.; Wang, C.; Liu, M.; Yin, B.; A, P.; Huang, D.; Ye, L. Inhibition of SOX9 Promotes Inflammatory and Immune Responses of Dental Pulp. *Journal of Endodontics* **2018**, *44*, 792-799, doi:10.1016/j.joen.2018.02.004.
41. Mahmoudi, J.; Sabermarouf, B.; Baradaran, B.; Sadat-Hatamnezhad, L.; Shotorbani, S.S. Up-regulation of TLR2 and TLR4 in high mobility group Box1-stimulated macrophages in pulpitis patients. *Iranian Journal of Basic Medical Sciences* **2017**, *20*, 209-215.
42. Meng, R.; Li, D.; Feng, Z.; Xu, Q. MyD88 hypermethylation mediated by DNMT1 is associated with LTA-induced inflammatory response in human odontoblast-like cells. *Cell and tissue research* **2019**, doi:10.1007/s00441-019-02993-0.
43. Min, K.S.; Kim, H.I.; Chang, H.S.; Kim, H.R.; Pae, H.O.; Chung, H.T.; Hong, S.H.; Shin, H.I.; Hong, C.U.; Lee, S.K.; et al. Involvement of mitogen-activated protein kinases and nuclear factor-kappa B activation in nitric oxide-induced interleukin-8 expression in human pulp cells. *Oral Surgery Oral Medicine Oral Pathology Oral Radiology & Endodontics* **2008**, *105*, 654-660, doi:https://dx.doi.org/10.1016/j.tripleo.2007.11.011.
44. Park, S.H.; Hsiao, G.Y.; Huang, G.T. Role of substance P and calcitonin gene-related peptide in the regulation of interleukin-8 and monocyte chemotactic protein-1 expression in human dental pulp. *International endodontic journal* **2004**, *37*, 185-192.
45. Pereira, L.O.; Rubini, M.R.; Silva, J.R.; Oliveira, D.M.; Silva, I.C.R.; Poças-Fonseca, M.J.; Azevedo, R.B. Comparison of stem cell properties of cells isolated from normal and inflamed dental pulps. **2012**, *45*, 1080-1090, doi:https://doi.org/10.1111/j.1365-2591.2012.02068.x.
46. Rhim, E.M.; Ahn, S.J.; Kim, J.Y.; Kim, K.H.; Lee, H.W.; Kim, E.C.; Kim, K.Y.; Park, S.H. Stimulation of Matrix Metalloproteinases by Tumor Necrosis Factor-alpha in Human Pulp Cell Cultures. *Journal of Endodontics* **2013**, *39*, 795-800, doi:10.1016/j.joen.2012.12.038.
47. Satrawaha, S.; Wongkhantee, S.; Pavasant, P.; Sumrejkanchanakij, P. Pressure induces interleukin-6 expression via the P2Y6 receptor in human dental pulp cells. *Archives of Oral Biology* **2011**, *56*, 1230-1237, doi:10.1016/j.archoralbio.2011.05.003.
48. Shindo, S.; Kumagai, T.; Shirawachi, S.; Takeda, K.; Shiba, H. Semaphorin3A released from human dental pulp cells inhibits the increase in interleukin-6 and CXC chemokine ligand 10 production induced by tumor necrosis factor-α through suppression of nuclear factor-κB activation. **2021**, *45*, 238-244, doi:https://doi.org/10.1002/cbin.11466.
49. Soares, D.G.; Zhang, Z.P.; Mohamed, F.; Eyster, T.W.; Costa, C.A.D.; Ma, P.X. Simvastatin and nanofibrous poly(L-lactic acid) scaffolds to promote the odontogenic potential of dental pulp cells in an inflammatory environment. *Acta Biomaterialia* **2018**, *68*, 190-203, doi:10.1016/j.actbio.2017.12.037.

50. Song, F.; Sun, H.; Wang, Y.; Yang, H.; Huang, L.; Fu, D.; Gan, J.; Huang, C. Pannexin3 inhibits TNF-alpha-induced inflammatory response by suppressing NF-kappaB signalling pathway in human dental pulp cells. *Journal of Cellular & Molecular Medicine* **2017**, *21*, 444-455, doi:https://dx.doi.org/10.1111/jcmm.12988.
51. Sugiuchi, A.; Sano, Y.; Furusawa, M.; Abe, S.; Muramatsu, T. Human Dental Pulp Cells Express Cellular Markers for Inflammation and Hard Tissue Formation in Response to Bacterial Information. *Journal of Endodontics* **2018**, *44*, 992-996, doi:10.1016/j.joen.2018.02.022.
52. Takanche, J.S.; Lee, Y.-H.; Kim, J.-S.; Kim, J.-E.; Han, S.-H.; Lee, S.-W.; Yi, H.-K. Anti-inflammatory and antioxidant properties of Schisandrin C promote mitochondrial biogenesis in human dental pulp cells. **2018**, *51*, 438-447, doi:https://doi.org/10.1111/iej.12861.
53. Takegawa, D.; Nakanishi, T.; Hirao, K.; Yumoto, H.; Takahashi, K.; Matsuo, T. Modulatory roles of interferon-gamma through indoleamine 2, 3-dioxygenase induction in innate immune response of dental pulp cells. *Journal of Endodontics* **2014**, *40*, 1382-1387, doi:https://dx.doi.org/10.1016/j.joen.2014.03.018.
54. Tamura, M.; Nagaoka, S.; Kawagoe, M. Interleukin-1 alpha stimulates interstitial collagenase gene expression in human dental pulp fibroblast. *Journal of Endodontics* **1996**, *22*, 240-243.
55. Teja, K.V.; Ramesh, S.; Priya, V. Regulation of matrix metalloproteinase-3 gene expression in inflammation: A molecular study. *J* **2018**, *21*, 592-596, doi:10.4103/JCD.JCD_154_18.
56. Tjaderhane, L.; Palosaari, H.; Sulkala, M.; Wahlgren, J.; Salo, T. *The expression of matrix metalloproteinases (MMPs) in human odontoblasts*; 2002; pp. 45-51.
57. Tokuda, M.; Sakuta, T.; Fushuku, A.; Torii, M.; Nagaoka, S. Regulation of interleukin-6 expression in human dental pulp cell cultures stimulated with Prevotella intermedia lipopolysaccharide. *Journal of Endodontics* **2001**, *27*, 273-277.
58. Tsai, A.I.; Hong, H.H.; Lin, W.R.; Fu, J.F.; Chang, C.C.; Wang, I.K.; Huang, W.H.; Weng, C.H.; Hsu, C.W.; Yen, T.H. Isolation of Mesenchymal Stem Cells from Human Deciduous Teeth Pulp. *BioMed research international* **2017**, *2017*, doi:10.1155/2017/2851906.
59. Wang, D.; Sun, S.; Xue, Y.; Qiu, J.; Ye, T.; Zhang, R.; Song, B.; He, W.; Zhang, Y.; Jiang, W. MicroRNA-223 negatively regulates LPS-induced inflammatory responses by targeting NLRP3 in human dental pulp fibroblasts. *International endodontic journal* **2021**, *54*, 241-254, doi:10.1111/iej.13413.
60. Wang, F.; Han, Y.; Xi, S.; Lu, Y. Catechins reduce inflammation in lipopolysaccharide-stimulated dental pulp cells by inhibiting activation of the NF-κB pathway. **2020**, *26*, 815-821, doi:https://doi.org/10.1111/odi.13290.
61. Wang, X.; Feng, Z.; Li, Q.; Yi, B.; Xu, Q. DNA methylcytosine dioxygenase ten-eleven translocation 2 enhances lipopolysaccharide-induced cytokine expression in human dental pulp cells by regulating MyD88 hydroxymethylation. *Cell and tissue research* **2018**, *373*, 477-485, doi:10.1007/s00441-018-2826-x.
62. Wei, L.; Liu, M.; Xiong, H.; Peng, B. Up-regulation of IL-23 expression in human dental pulp fibroblasts by IL-17 via activation of the NF-κB and MAPK pathways. **2018**, *51*, 622-631, doi:https://doi.org/10.1111/iej.12871.
63. Widbiller, M.; Eidt, A.; Wölflick, M.; Lindner, S.R.; Schweikl, H.; Hiller, K.A.; Buchalla, W.; Galler, K.M. Interactive effects of LPS and dentine matrix proteins on human dental pulp stem cells. *International endodontic journal* **2018**, *51*, 877-888, doi:10.1111/iej.12897.

64. Wisithphrom, K.; Windsor, L.J. The effects of tumor necrosis factor-alpha, interleukin-1 beta, interleukin-6, and transforming growth factor-beta 1 on pulp fibroblast mediated collagen degradation. *Journal of Endodontics* **2006**, *32*, 853-861, doi:10.1016/j.joen.2006.03.017.
65. Xue, D.; Gong, Z.; Zhu, F.; Qiu, Y.; Li, X. Simvastatin increases cell viability and suppresses the expression of cytokines and vascular endothelial growth factor in inflamed human dental pulp stem cells in vitro. *Advances in Clinical and Experimental Medicine* **2018**, *27*, 1615-1623, doi:10.17219/acem/75776.
66. Yamada, Y.; Nakamura-Yamada, S.; Umemura-Kubota, E.; Baba, S. Diagnostic cytokines and comparative analysis secreted from exfoliated deciduous teeth, dental pulp, and bone marrow derived mesenchymal stem cells for functional cell-based therapy. *International Journal of Molecular Sciences* **2019**, *20*, doi:10.3390/ijms20235900.
67. Yang, G.; Ju, Y.; Liu, S.; Zhao, S. Lipopolysaccharide upregulates the proliferation, migration, and odontoblastic differentiation of NG2+ cells from human dental pulp in vitro. **2019**, *43*, 1276-1285, doi:https://doi.org/10.1002/cbin.11127.
68. Yang, L.-C.; Tsai, C.-H.; Huang, F.-M.; Liu, C.-M.; Lai, C.-C.; Chang, Y.-C. Induction of interleukin-6 gene expression by pro-inflammatory cytokines and black-pigmented Bacteroides in human pulp cell cultures. **2003**, *36*, 352-357, doi:https://doi.org/10.1046/j.1365-2591.2003.00663.x.
69. Yonehiro, J.; Yamashita, A.; Yoshida, Y.; Yoshizawa, S.; Ohta, K.; Kamata, N.; Okihara, T.; Nishimura, F. Establishment of an ex vivo pulpitis model by co-culturing immortalized dental pulp cells and macrophages. **2012**, *45*, 1103-1108, doi:https://doi.org/10.1111/j.1365-2591.2012.02074.x.
70. Yu, M.K.; Lee, J.C.; Kim, J.H.; Lee, Y.H.; Jeon, J.G.; Jhee, E.C.; Yi, H.K. Anti-inflammatory Effect of Peroxisome Proliferator Activated Receptor Gamma on Human Dental Pulp Cells. *Journal of Endodontics* **2009**, *35*, 524-528, doi:10.1016/j.joen.2008.12.012.
71. Yu, S.; Diao, S.; Wang, J.; Ding, G.; Yang, D.; Fan, Z. Comparative analysis of proliferation and differentiation potentials of stem cells from inflamed pulp of deciduous teeth and stem cells from exfoliated deciduous teeth. *BioMed research international* **2014**, *2014*, doi:10.1155/2014/930907.
72. Zhai, S.; Wang, Y.; Jiang, W.; Jia, Q.; Li, J.; Wang, W.; Wang, H.; Ding, Y.; Wang, P.; Liu, J.; et al. Nemotic human dental pulp fibroblasts promote human dental pulp stem cells migration. *Experimental cell research* **2013**, *319*, 1544-1552, doi:10.1016/j.yexcr.2013.03.018.
73. Zhai, Y.; Wang, Y.; Rao, N.; Li, J.; Li, X.; Fang, T.; Zhao, Y.; Ge, L. Activation and Biological Properties of Human β Defensin 4 in Stem Cells Derived From Human Exfoliated Deciduous Teeth. *Frontiers in Physiology* **2019**, *10*, doi:10.3389/fphys.2019.01304.
74. Zhai, Y.; Yuan, X.; Zhao, Y.; Ge, L.; Wang, Y. Potential Application of Human β-Defensin 4 in Dental Pulp Repair. *Frontiers in Physiology* **2020**, *11*, doi:10.3389/fphys.2020.01077.
75. Zhao, Y.; Wang, C.L.; Li, R.M.; Hui, T.Q.; Su, Y.Y.; Yuan, Q.; Zhou, X.D.; Ye, L. Wnt5a promotes inflammatory responses via nuclear factor kappaB (NF-kappaB) and mitogen-activated protein kinase (MAPK) pathways in human dental pulp cells. *Journal of Biological Chemistry* **2014**, *289*, 21028-21039.

76. Lei, F.; Zhang, H.; Xie, X. Comprehensive analysis of an lncRNA-miRNA-mRNA competing endogenous RNA network in pulpitis. *PeerJ* **2019**, *2019*, doi:10.7717/peerj.7135.
77. Bruno, K.F.; Silva, J.A.; Silva, T.A.; Batista, A.C.; Alencar, A.H.G.; Estrela, C. Characterization of inflammatory cell infiltrate in human dental pulpitis. **2010**, *43*, 1013-1021, doi:https://doi.org/10.1111/j.1365-2591.2010.01757.x.
78. Giorgini, E.; Sabbatini, S.; Conti, C.; Rubini, C.; Rocchetti, R.; Fioroni, M.; Memè, L.; Orilisi, G. Fourier Transform Infrared Imaging analysis of dental pulp inflammatory diseases. **2017**, *23*, 484-491, doi:https://doi.org/10.1111/odi.12635.
79. Anshida, V.P.; Kumari, R.A.; Murthy, C.S.; Samuel, A. Extracellular matrix degradation by host matrix metalloproteinases in restorative dentistry and endodontics: An overview. *J* **2020**, *24*, 352-360, doi:https://dx.doi.org/10.4103/jomfp.JOMFP_34_20.
80. Arora, S.; Cooper, P.R.; Friedlander, L.T.; Rizwan, S.; Seo, B.; Rich, A.M.; Hussaini, H.M. Potential application of immunotherapy for modulation of pulp inflammation: opportunities for vital pulp treatment. *n/a*, doi:https://doi.org/10.1111/iej.13524.
81. Emilia, E.; Neelakantan, P. Biomarkers in the dentin-pulp complex: role in health and disease. *The Journal of clinical pediatric dentistry* **2015**, *39*, 94-99, doi:10.17796/jcpd.39.2.r32617516412p710.
82. Hirsch, V.; Wolgin, M.; Mitronin, A.V.; Kielbassa, A.M. Inflammatory cytokines in normal and irreversibly inflamed pulps: A systematic review. *Archives of Oral Biology* **2017**, *82*, 38-46, doi:10.1016/j.archoralbio.2017.05.008.
83. Khorasani, M.M.Y.; Hassanshahi, G.; Brodzikowska, A.; Khorramdelazad, H. Role(s) of cytokines in pulpitis: Latest evidence and therapeutic approaches. *Cytokine* **2020**, *126*, doi:10.1016/j.cyto.2019.154896.
84. Nibali, L.; Fedele, S.; D'Aiuto, F.; Donos, N. Interleukin-6 in oral diseases: a review. **2012**, *18*, 236-243, doi:https://doi.org/10.1111/j.1601-0825.2011.01867.x.
85. Rechenberg, D.K.; Galicia, J.C.; Peters, O.A. Biological markers for pulpal inflammation: A systematic review. *PloS one* **2016**, *11*, doi:10.1371/journal.pone.0167289.
86. Sambandam, V.; Neelakantan, P. Matrix Metalloproteinases (Mmp) in Restorative Dentistry and Endodontics. *Journal of Clinical Pediatric Dentistry* **2014**, *39*, 57-59, doi:10.17796/jcpd.39.1.x452446251r1q428.
87. Zanini, M.; Meyer, E.; Simon, S. Pulp Inflammation Diagnosis from Clinical to Inflammatory Mediators: A Systematic Review. *Journal of Endodontics* **2017**, *43*, 1033-1051, doi:10.1016/j.joen.2017.02.009.
88. Accorsi-Mendonça, T.; Silva, E.J.N.L.; Marcaccini, A.M.; Gerlach, R.F.; Duarte, K.M.R.; Pardo, A.P.S.; Line, S.R.P.; Zaia, A.A. Evaluation of gelatinases, tissue inhibitor of matrix metalloproteinase-2, and myeloperoxidase protein in healthy and inflamed human dental pulp tissue. *Journal of Endodontics* **2013**, *39*, 879-882, doi:10.1016/j.joen.2012.11.011.
89. Alvarez, M.M.P.; Moura, G.E.; Machado, M.F.M.; Viana, G.M.; de Souza Costa, C.A.; Tjäderhane, L.; Nader, H.B.; Tersariol, I.L.S.; Nascimento, F.D. PAR-1 and PAR-2 Expression Is Enhanced in Inflamed Odontoblast Cells. *Journal of dental research* **2017**, *96*, 1518-1525, doi:10.1177/0022034517719415.

90. Brodzikowska, A.; Gondek, A.; Rak, B.; Paskal, W.; Pełka, K.; Cudnoch-Jędrzejewska, A.; Włodarski, P. Metalloproteinase 14 (MMP-14) and hsa-miR-410-3p expression in human inflamed dental pulp and odontoblasts. *Histochemistry and Cell Biology* **2019**, *152*, 345-353, doi:10.1007/s00418-019-01811-6.
91. Galicia, J.C.; Henson, B.R.; Parker, J.S.; Khan, A.A. Gene expression profile of pulpitis. *Genes and Immunity* **2016**, *17*, 239-243, doi:10.1038/gene.2016.14.
92. Gatta, V.; Zizzari, V.L.; Dd ' Amico, V.; Salini, L.; M, D.A.; Franchi, S.; Antonucci, I.; Sberna, M.T.; Gherlone, E.; Stuppia, L.; et al. Microarray evaluation of gene expression profiles in inflamed and healthy human dental pulp: the role of IL1beta and CD40 in pulp inflammation. *Journal of biological regulators and homeostatic agents* **2012**, *26*, 45-50.
93. Liu, M.; Zhao, Y.; Wang, C.; Luo, H.; A, P.; Ye, L. Interleukin-17 plays a role in pulp inflammation partly by WNT5A protein induction. *Arch Oral Biol* **2019**, *103*, 33-39, doi:10.1016/j.archoralbio.2019.05.003.
94. Tsai, C.H.; Chen, Y.J.; Huang, F.M.; Su, Y.F.; Chang, Y.C. The upregulation of matrix metalloproteinase-9 in inflamed human dental pulps. *Journal of Endodontics* **2005**, *31*, 860-862.
95. Xiong, H.; Wei, L.; Peng, B. IL-17 stimulates the production of the inflammatory chemokines IL-6 and IL-8 in human dental pulp fibroblasts. **2015**, *48*, 505-511, doi:https://doi.org/10.1111/iej.12339.
96. Zehnder, M.; Delaleu, N.; Du, Y.; Bickel, M. Cytokine gene expression - Part of host defence in pulpitis. *Cytokine* **2003**, *22*, 84-88, doi:10.1016/S1043-4666(03)00116-9.
97. Zhang, N.; Zhang, Q.; Yang, W.; Miao, L.; Wang, N.; Wei, S.; Ge, J.; Li, X.; Wu, J. Decreased expression of microRNA-30b promotes the development of pulpitis by upregulating the expression of interleukin-6 receptor. *Experimental and therapeutic medicine* **2019**, *17*, 3233-3238, doi:10.3892/etm.2019.7280.
98. Aishuwariya, T.; Ramesh, S. Evaluation of interleukin 6, interleukin 8, tnf alpha as biomarkers for pulpitis-in vivo study. *International Journal of Dentistry and Oral Science* **2021**, *8*, 1492-1497.
99. Ballal, V.; Rao, S.; Bagheri, A.; Bhat, V.; Attin, T.; Zehnder, M. MMP-9 in Dentinal Fluid Correlates with Caries Lesion Depth. *Caries research* **2017**, *51*, 460-465, doi:10.1159/000479040.
100. Brizuela, C.; Meza, G.; Mercadé, M.; Inostroza, C.; Chaparro, A.; Bravo, I.; Briceño, C.; Hernández, M.; Giner, L.; Ramírez, V. Inflammatory biomarkers in dentinal fluid as an approach to molecular diagnostics in pulpitis. **2020**, *53*, 1181-1191, doi:https://doi.org/10.1111/iej.13343.
101. ElSalhy, M.; Azizieh, F.; Raghupathy, R. Cytokines as diagnostic markers of pulpal inflammation. **2013**, *46*, 573-580, doi:https://doi.org/10.1111/iej.12030.
102. Mente, J.; Petrovic, J.; Gehrig, H.; Rampf, S.; Michel, A.; Schürz, A.; Pfefferle, T.; Saure, D.; Erber, R. A Prospective Clinical Pilot Study on the Level of Matrix Metalloproteinase-9 in Dental Pulpal Blood as a Marker for the State of Inflammation in the Pulp Tissue. *Journal of Endodontics* **2016**, *42*, 190-197, doi:10.1016/j.joen.2015.10.020.

103. Nakanishi, T.; Matsuo, T.; Ebisu, S. Quantitative analysis of immunoglobulins and inflammatory factors in human pulpal blood from exposed pulps. *Journal of Endodontics* **1995**, *21*, 131-136, doi:10.1016/S0099-2399(06)80438-3.
104. Rechenberg, D.K.; Bostanci, N.; Zehnder, M.; Belibasakis, G.N. Periapical fluid RANKL and IL-8 are differentially regulated in pulpitis and apical periodontitis. *Cytokine* **2014**, *69*, 116-119, doi:10.1016/j.cyto.2014.05.014.
105. Sharma, R.; Kumar, V.; Logani, A.; Chawla, A.; Mir, R.A.; Sharma, S.; Kalaivani, M. Association between concentration of active MMP-9 in pulpal blood and pulpotomy outcome in permanent mature teeth with irreversible pulpitis – a preliminary study. **2021**, *54*, 479-489, doi:https://doi.org/10.1111/iej.13437.
106. Shimada, Y.; Ichinose, S.; Sadr, A.; Burrow, M.; Tagami, J. Localization of matrix metalloproteinases (MMPs-2, 8, 9 and 20) in normal and carious dentine. **2009**, *54*, 347-354, doi:https://doi.org/10.1111/j.1834-7819.2009.01161.x.
107. Zehnder, M.; Wegehaupt, F.J.; Attin, T. A first study on the usefulness of matrix metalloproteinase 9 from dentinal fluid to indicate pulp inflammation. *Journal of Endodontics* **2011**, *37*, 17-20, doi:10.1016/j.joen.2010.10.003.
108. Zhu, Z.y.; Zhou, T.; Zhang, B.w. Characterization and analysis of matrix metalloproteinases 8 and 20 in the human crown and root dentin. *Chinese Journal of Tissue Engineering Research* **2012**, *16*, 4526-4529, doi:10.3969/j.issn.1673-8225.2012.24.032.
109. Wahlgren, J.; Salo, T.; Teronen, O.; Luoto, H.; Sorsa, T.; Tjäderhane, L. Matrix metalloproteinase-8 (MMP-8) in pulpal and periapical inflammation and periapical root-canal exudates. *International endodontic journal* **2002**, *35*, 897-904, doi:10.1046/j.1365-2591.2002.00587.x.
110. Zhou, S.; Xu, J. Downregulation of microRNA-204 increases the expression of matrix metallopeptidase 9 in pediatric patients with pulpitis and Helicobacter pylori infection in the stomach. *Experimental and therapeutic medicine* **2019**, *18*, 253-259, doi:10.3892/etm.2019.7528.
111. Anderson, L.M.; Dumsha, T.C.; McDonald, N.J.; Spitznagel Jr, J.K. Evaluating iL-2 levels in human pulp tissue. *Journal of Endodontics* **2002**, *28*, 651-655, doi:10.1097/00004770-200209000-00006.
112. Barkhordar, R.A.; Hayashi, C.; Hussain, M.Z. Detection of interleukin-6 in human dental pulp and periapical lesions. **1999**, *15*, 26-27, doi:https://doi.org/10.1111/j.1600-9657.1999.tb00744.x.
113. Gusman, H.; Santana, R.B.; Zehnder, M. Matrix metalloproteinase levels and gelatinolytic activity in clinically healthy and inflamed human dental pulps. **2002**, *35*, 897-904, doi:https://doi.org/10.1046/j.1365-2591.2002.00587.x.
114. Huang, G.T.J.; Potente, A.P.; Kim, J.W.; Chugal, N.; Zhang, X. Increased interleukin-8 expression in inflamed human dental pulps. *Oral surgery, oral medicine, oral pathology, oral radiology, and endodontics* **1999**, *88*, 214-220, doi:10.1016/S1079-2104(99)70118-6.
115. Rauschenberger, C.R.; Bailey, J.C.; Cootauco, C.J. Detection of human IL-2 in normal and inflamed dental pulps. *Journal of Endodontics* **1997**, *23*, 366-370.
116. Sattari, M.; Haghighi, A.K.; Tamijani, H.D. The relationship of pulp polyp with the presence and concentration of immunoglobulin E, histamine, interleukin-4 and interleukin-12. **2009**, *35*, 164-168, doi:https://doi.org/10.1111/j.1747-4477.2009.00160.x.

117. Shin, S.J.; Lee, J.I.; Baek, S.H.; Lim, S.S. Tissue levels of matrix metalloproteinases in pulps and periapical lesions. *Journal of Endodontics* **2002**, *28*, 313-315, doi:10.1097/00004770-200204000-00013.
118. Silva, A.C.; Faria, M.R.; Fontes, A.; Campos, M.S.; Cavalcanti, B.N. Interleukin-1 beta and interleukin-8 in healthy and inflamed dental pulps. *J* **2009**, *17*, 527-532.
119. Abd-Elmeguid, A.; Yu, D.C.; Kline, L.W.; Moqbel, R.; Vliagoftis, H. Dentin matrix protein-1 activates dental pulp fibroblasts. *Journal of Endodontics* **2012**, *38*, 75-80, doi:10.1016/j.joen.2011.10.005.
120. Abd-Elmeguid, A.; Abdeldayem, M.; Kline, L.W.; Moqbel, R.; Vliagoftis, H.; Yu, D.C. Osteocalcin expression in pulp inflammation. *Journal of Endodontics* **2013**, *39*, 865-872, doi:10.1016/j.joen.2012.12.035.
121. Akbal Dincer, G.; Erdemir, A.; Kisa, U. Comparison of Neurokinin A, Substance P, Interleukin 8, and Matrix Metalloproteinase-8 Changes in Pulp tissue and Gingival Crevicular Fluid Samples of Healthy and Symptomatic Irreversible Pulpitis Teeth. *Journal of Endodontics* **2020**, *46*, 1428-1437, doi:10.1016/j.joen.2020.07.013.
122. Evrosimovska, B.; Dimova, C.; Kovacevska, I.; Panov, S. Concentration of collagenases (MMP-1, -8, -13) in patients with chronically inflamed dental pulp tissue. *Prilozi / Makedonska akademija na naukite i umetnostite, Oddelenie za biološki i medicinski nauki = Contributions / Macedonian Academy of Sciences and Arts, Section of Biological and Medical Sciences* **2012**, *33*, 191-204.
123. Šubarić, L.; Mitić, A.; Matvijenko, V.; Jovanović, R.; Živković, D.; Perić, D.; Vlahović, Z. Interleukin 1-beta analysis in chronically inflamed and healthy human dental pulp. *Vojnosanitetski Pregled* **2017**, *74*, 256-260, doi:10.2298/VSP140303172S.
124. Suwanchai, A.; Theerapiboon, U.; Chattipakorn, N.; Chattipakorn, S.C. NaV1.8, but not NaV1.9, is upregulated in the inflamed dental pulp tissue of human primary teeth. **2012**, *45*, 372-378, doi:https://doi.org/10.1111/j.1365-2591.2011.01986.x.
125. Page, M.J.; McKenzie, J.E.; Bossuyt, P.M.; Boutron, I.; Hoffmann, T.C.; Mulrow, C.D.; Shamseer, L.; Tetzlaff, J.M.; Akl, E.A.; Brennan, S.E.; et al. The PRISMA 2020 statement: An updated guideline for reporting systematic reviews. *Int J Surg* **2021**, *88*, 105906, doi:10.1016/j.ijsu.2021.105906.
